# Exploring a Water–Ethyl Acetate System for the Efficient Synthesis of 4‐Aryl Quinolines

**DOI:** 10.1002/open.202400470

**Published:** 2025-04-07

**Authors:** Mohammad Qandalee, Aliyeh Khajeh‐Khezri, Mohammad Alikarami, Silvia Izquierdo, Ignacio M. López‐Coca

**Affiliations:** ^1^ Department of Basic Sciences Garmsar Branch Islamic Azad University Garmsar 3581631167 Iran; ^2^ Department of Chemistry Tarbiat Modares University Tehran 14115111 Iran; ^3^ Department of Chemistry Ilam Branch Islamic Azad University Ilam 6931133145 Iran; ^4^ Department of Organic and Inorganic Chemistry School of Technology INTERRA Universidad de Extremadura Cáceres 10003 Spain

**Keywords:** arylquinolines, catalyst‐free reactions, heterocycles, organic electronic devices

## Abstract

Heterocyclic compounds, particularly aryl quinolines, are of paramount importance in pharmaceuticals, materials science, and agrochemicals owing to their diverse biological and physicochemical properties. However, traditional synthesis methods often rely on organic solvents and catalysts, which raise environmental and health concerns while requiring high energy inputs. This study addresses these challenges by applying the principles of green chemistry to develop a sustainable, efficient, and environmentally friendly synthetic route for aryl quinolines.

## Graphical abstract

A sustainable and catalyst‐free method for synthesizing 4‐aryl quinolines is developed using a water–ethyl acetate solvent system under reflux conditions. This approach eliminates hazardous reagents while achieving high yields and purity. The protocol aligns with green chemistry principles, offering an efficient and environmentally friendly alternative for the synthesis of quinoline derivatives.

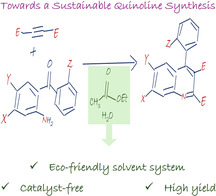

## 1 Introduction

**Figure 1:**
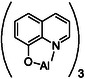
Tris‐(8‐hydroxyquinoline) aluminum (AlQ_3_).

Owing to their wide application in industry and medicine, quinoline‐containing scaffolds have garnered significant interest from researchers seeking new strategies for producing this class of heterocyclic materials. These compounds exhibit diverse biological activities, including antibacterial, antiplatelet, antihypertensive, anti‐inflammatory, and antimalarial properties.^[^ ^1^, ^2^, ^3^, ^4^, ^5^, ^6^, ^7^, ^8^, ^9^, ^10^, ^11^ ^]^ Quinolines have been widely investigated for their potential in various therapeutic applications. Furthermore, the versatile nature of quinolines extends beyond medicinal uses, finding relevance in organic electronic devices, particularly metalquinolates. Notably, tris‐(8‐hydroxyquinoline)‐aluminum (AlQ3, **Figure** **1**) has emerged as a prominent electron‐transporting and emitting material in the manufacture of organic light‐emitting diodes (OLEDs).^[^ ^12^, ^13^, ^14^ ^]^ Metalquinolates, including AlQ_3_, have become standard electroluminescent compounds for OLEDs since 1987, representing a well‐studied and established system.^[^ ^15^, ^16^, ^17^ ^]^ Given these applications, there is a continuing need to develop novel materials to further advance the field of OLED systems.

Due to the importance and versatility of quinolines, numerous classical synthetic routes have been developed to access these valuable compounds. Examples of well‐known methods include the Combes and Doebner–von Miller,^[^ ^18^ ^]^ Skraup and Friedlander,^[^ ^19^ ^]^ Pfitzinger,^[^ ^20^ ^]^ and Conrad–Limpach reactions.^[^ ^21^ ^]^ These traditional approaches have been extensively reviewed, providing a solid foundation for synthesizing quinoline‐based compounds.^[^ ^22^ ^]^ Recently, research has focused on exploring innovative strategies to access quinoline derivatives. For instance, a previously reported Zn‐based metal‐organic framework has been utilized as a catalyst in the synthesis of 2,4‐disubstituted quinoline derivatives with promising results.^[^ ^23^ ^]^ Additionally, a catalyst‐free method employing dimethyl sulfoxide (DMSO) as a solvent has shown efficacy in synthesizing 2,3‐dicarboxylic ester quinoline derivatives.^[^ ^24^ ^]^ These advancements contribute to expanding the repertoire of synthetic methodologies for quinoline systems, enabling the development of diverse chemical libraries.

The field of green chemistry provides a holistic approach to designing and optimizing chemical processes with minimal environmental impact. By focusing on the principles of waste prevention, atom economy, and energy efficiency, green chemistry aims to minimize the generation of hazardous waste, reduce resource consumption, and decrease the use of toxic solvents and catalysts. Homogeneous and heterogeneous catalysts are commonly employed in traditional quinoline synthesis, but they often pose challenges in terms of separation, reusability, and toxicity. Green chemistry endeavors to develop catalyst‐free protocols, thereby eliminating the need for these potentially hazardous materials. The application of green chemistry principles to the synthesis of aryl quinolines has garnered significant attention in recent years to develop environmentally benign and economically viable routes.^[^ ^25^, ^26^ ^]^ Recent research has explored innovative methodologies for the synthesis of quinolines, with a focus on efficiency and sustainability. A copper‐catalyzed one‐pot cascade reaction has been developed for the synthesis of 3‐ketoquinolines from saturated ketones and anthranils via sequential dehydrogenation, aza‐Michael addition, and annulation.^[^ ^27^ ^]^ Additionally, a transition‐metal‐free approach utilizing DMSO as both a solvent and a one‐carbon source has been reported for the synthesis of 3‐ketoquinolines from acetophenones and anthranils.^[^ ^28^ ^]^ Another metal‐free strategy has enabled the synthesis of 4‐aryl quinolines through a cascade process involving alkynes, anilines, and DMSO, demonstrating broad substrate scope and synthetic utility.^[^ ^29^ ^]^ These studies highlight the growing focus on environmentally benign and efficient strategies for quinoline synthesis, aligning with the principles of green chemistry.

Solvent selection in green synthesis plays a pivotal role in chemical reactions, affecting reaction rates, selectivity, and the overall environmental impact of the process. The choice of solvent has a significant effect on energy consumption, waste generation, and the toxicity profile of a given synthetic protocol. Green chemistry principles emphasize the use of benign and sustainable solvents, such as water and ethanol, as well as bio‐based alternatives, while seeking to minimize or eliminate reliance on volatile organic solvents. Recent studies have demonstrated the feasibility of solvent‐free or solvent‐minimized approaches for synthesizing aryl quinolines, offering a more environmentally friendly alternative and reducing the ecological footprint of these processes.^[^ ^30^ ^]^

As part of our ongoing efforts to contribute to the development of new methods for quinoline systems and their applications in various industries,^[^ ^31^, ^32^ ^]^ we report herein a simple and facile catalyst‐free reaction for synthesizing a series of quinoline derivatives. Specifically, we describe the synthesis of these derivatives through the reaction between 2‐amino benzophenone derivatives and acetylenic mono‐ or di‐carboxylate esters, employing a water–ethyl acetate (20:80) solvent system under reflux conditions for 1 h. Notably, the quinoline rings in the synthesized compounds were previously accessed using more stringent requirements in three‐component reactions. However, during our investigation into the synthesis of quinolines for electronic purposes, we discovered a new, eco‐friendly, and cost‐effective route to produce quinolines under milder and simpler reaction conditions. This method offers improved sustainability, reduced reaction complexity, and potential scalability. The detailed results of our electronic research will be presented separately, highlighting the exciting potential of these synthesized quinoline derivatives in OLED systems.

In summary, quinoline‐containing compounds have emerged as valuable building blocks due to their wide‐ranging applications in industry and medicine. The exploration of novel synthetic methodologies and the development of new quinoline derivatives have provided researchers with enhanced tools to unlock the full potential of these heterocyclic scaffolds. The catalyst‐free reaction described in this study represents a significant step forward in pursuing efficient, sustainable, and cost‐effective quinoline synthesis. It holds great promise for discovering novel materials and their applications in various fields, including OLED technology.

## 2 Results and Discussion

**Scheme 1:**
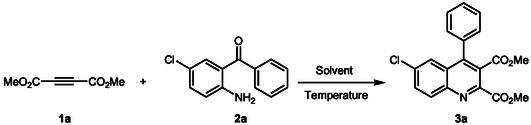
Synthesis of dimethyl 6‐chloro‐4‐phenylquinoline‐2,3‐dicarboxylate (**3a**).

Optimization of chemical reactions plays a pivotal role in developing efficient and cost‐effective synthetic methodologies. Our study chose the reaction between dimethyl acetylenedicarboxylate (DMAD) (**1a**) and 2‐amino‐5‐chlorobenzophenone (**2a**) as a model reaction (**Scheme** **1**). Selecting an appropriate reaction medium is vital for successful organic synthesis. Consequently, **Table** **1** presents the results of our optimization process for synthesizing aryl quinoline **3a**. Initially, we explored the effects of various solvents, including dichloromethane (CH_2_Cl_2_), toluene, benzene, water, and different ratios of water and ethyl acetate (EtOAc). The temperature of the reactions ranged from room temperature (r.t.) to reflux, resulting in varying reaction times.

**Table 1:** Optimization of the reaction conditions for the preparation of product 3a.

Entrya)	Solvent	Temp [°C]	Time [h]	Yieldb) [%]
1	CH_2_Cl_2_	r.t	10	10
2	Toluene	r.t	6	35
3	Benzene	r.t	10	7
4	H_2_O	r.t	12	0
5	H_2_O: EtOAc (20:80)	r.t	3	75
6	H_2_O: EtOAc (50:50)	r.t	10	20
7	H_2_O: EtOAc (20:80)	80	1	92
8	CH_2_Cl_2_	40	3	55
9	Toluene	110	3	64
10	Benzene	80	3	35
11	H_2_O	100	24	0
12	EtOAc	77	1	35

a) Reaction conditions: **1a** (1 mmol), **2a** (1 mmol), solvent (5 mL).

b) Isolated yield.

Entry 1 utilized dichloromethane as the solvent at r.t. for 10 h, yielding 10%. Entry 2 employed toluene as the solvent at r.t. for 6 h, leading to an improved yield of 35%. Toluene may have provided better solubility and stability for the reaction intermediates, thus enhancing the outcome compared to the previous entry. Entry 3 utilized benzene as the solvent at r.t. for 10 h, yielding a significantly lower result of 7%. In Entry 4, water was used as the solvent at r.t. for 12 h, but no product formation was observed. Water may either favor or hinder the reaction, depending on the desired product's formation. Entries 5 and 6 employed a mixture of water and ethyl acetate (H_2_O:EtOAc) at different ratios at r.t. for 3 and 10 h, respectively. Entry 5, with a ratio of 20:80 of H_2_O:EtOAc, yielded a remarkable 75% of the target compound. This result indicates that a moderate amount of water in the reaction mixture promotes the desired transformation. However, when the water content was increased to 50:50 in Entry 6, the yield decreased to 20%, indicating that excessive water might hinder the reaction or lead to side reactions. Entry 7 employed a 20:80 H_2_O:EtOAc ratio for 1 h at 80 °C, yielding an excellent 92% yield. Entries 8–11 used reflux conditions with different solvents (dichloromethane, toluene, benzene, and water) for 3 or 24 h. These entries exhibited varying yields, ranging from none to 64%. It is worth noting that using water as a solvent under reflux conditions (Entry 11) did not yield any product, further supporting the observation that water might not be suitable for this specific reaction. It is worth mentioning that this reaction has been carried out with 2‐amino benzophenone and DMAD, but no product has been produced at r.t. or after 24 h of reflux. Overall, the optimization results demonstrate the significant influence of reaction conditions on the yield of product **3a**. Finally, the reaction was carried out in neat ethyl acetate to check its performance as a solvent (Entry 12). The results demonstrate the importance of solvent choice, temperature, and reaction time in determining the reaction outcome. The optimized conditions, specifically a water–ethyl acetate ratio of 20:80 at 80 °C, demonstrated superior yields and promise for the efficient and scalable synthesis of product **3a**.

**Scheme 2:**
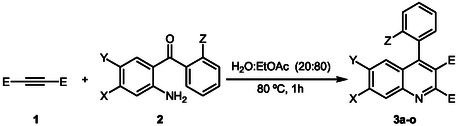
Synthesis of 4‐aryl quinoline derivatives from 2‐amino benzophenone derivatives and acetylenic esters.

After optimizing the reaction conditions, we investigated the scope of this reaction with various 2‐amino benzophenones and acetylenic esters (**Scheme** **2**). These results are summarized in **Table** **2**. Purification of quinolines **3a–o** was performed by simple crystallization from diethyl ether.

**Table 2:** Synthesis of 4‐aryl quinoline derivatives 3a–o.

Entry	Ea)	*X*	*Y*	*Z*	Product	Yieldb) [%]
1	CO_2_Me	H	Cl	H	**3a**	92
2	CO_2_Me	H	H	H	**3b**	92
3	CO_2_Me	H	Cl	Cl	**3c**	81
4	CO_2_Me	H	Cl	F	**3d**	94
5	CO_2_Me	CH_3_	H	H	**3e**	89
6	CO_2_Et	H	Cl	H	**3f**	93
7	CO_2_Et	H	H	H	**3g**	92
8	CO_2_Et	H	Cl	Cl	**3h**	89
9	CO_2_Et	H	Cl	F	**3i**	95
10	CO_2_Et	CH_3_	H	H	**3j**	91
11	CO_2_^*t*^Bu	H	Cl	Cl	**3k**	77
12	CO_2_^*t*^Bu	H	Cl	F	**3l**	85
13	CO_2_^*t*^Bu	CH_3_	H	H	**3m**	79
14	CO_2_^*t*^Bu	H	H	H	**3n**	92
15	CO_2_^*t*^Bu	H	Cl	H	**3o**	90

a) Reaction conditions: **1** (2 mmol), **2** (2 mmol), H_2_O:EtOAc 20:80 (10 mL), 1 h, 80 °C.

b) Isolated yield.

After optimizing the reaction conditions, the investigation focused on exploring the reaction scope between 2‐amino‐benzophenones and acetylenic diesters to synthesize 4‐aryl quinoline derivatives. The variables studied included variations in the substituents on the reactants, such as different ester groups (methyl, ethyl, and *tert*‐butyl) and substitutions on the benzophenone moiety (chlorine, fluorine, and methyl group) (Scheme 2). These results are summarized in Table 2.

The results demonstrate the reaction's versatility and provide valuable insights into the effects of different substituents on product formation. Globally, we can observe that the yields are good or excellent. It should be noted that the purification of the quinolines was carried out by crystallization in diethyl ether, and the isolated yield, ranging from 77% to 95%, is not optimized. Observing the reactions of the methyl diester, Entries 1–5, which yield 81–94%, we can conclude that the scope of the protocol is not limited by the presence of one or two halogens, nor by their absence, since the outcome of all reactions is similarly excellent. The same conclusion can be drawn from the reactions with the diethyl ester; Entries 6–10 yield 89–95% yields, as the results are consistent and very similar to those mentioned. When the bulkier di‐*tert*‐butyl ester is used (Entries 11–15), the yields of some products slightly decrease (Entry 11, 77%; Entry 13, 79%). This decrease is not attributable to the benzophenone substitution pattern; the explanation could be due to the ease with which these two products crystallized. The remaining reactions achieve a satisfactory yield of 85%–92%. This set of experiments illustrates the effect of the ester group in the acetylenic reactant and that of the substituents on the acetophenone molecule on the reaction outcome. It highlights the flexibility of the process to accommodate different ester functionalities and benzophenone substituents. The results obtained with the tertiary butyl ester show that this protocol also exhibits high tolerance to bulkier groups in the ester.

To assess the scope of this protocol, we conducted additional experiments using acetylenic monoesters, such as methyl and ethyl propiolate, as well as the unactivated alkyne, phenylacetylene, and in combination with various benzophenone derivatives, including 2‐amino benzophenone, 2‐amino‐5‐chlorobenzophenone, and 2‐amino‐4‐methylbenzophenone. These reactions were performed under the same conditions previously applied to acetylene diesters. However, no product formation was observed within the monitored timeframe, indicating that monoesters lack sufficient reactivity in this system.

To further investigate and potentially expand the substrate scope, we explored the reactivity of methyl propiolate and phenylacetylene with 2‐amino‐5‐chloro‐2’‐fluorobenzophenone in the presence of various copper and iron catalysts (10–20 mol%), including CuO, Cu(NO_3_)_2_, CuSO_4_, CuBr, and FeSO_4_. Despite these modifications, no reaction occurred in any case.

This lack of reactivity may be attributed to the lower electrophilic activation of these alkynes compared to acetylene diesters, which benefit from the strong electron‐withdrawing effect of two ester groups, thereby facilitating the transformation. Our findings suggest that the presence of a second ester is crucial for promoting the reaction. While copper and iron catalysts are known to mediate certain alkyne transformations, they did not induce the desired reactivity under our conditions.

Overall, the results demonstrate that this reaction exhibits a broad scope, enabling the efficient synthesis of diverse 4‐aryl quinoline derivatives. Notably, our methodology utilizes a solvent system comprising ethyl acetate and water. In contrast, a previously reported approach for quinoline synthesis utilized DMSO as the solvent, operating at 50 °C or 80 °C for 24 h without the use of a catalyst.^[^ ^24^ ^]^ According to Byrne et al. water and ethyl acetate are classified as environmentally preferred solvents, whereas DMSO is considered problematic due to concerns regarding its disposal and toxicity.^[^ ^33^ ^]^ By adopting greener solvents and significantly reducing reaction times, our protocol offers a more sustainable and efficient alternative, aligning with the principles of green chemistry.

**Scheme 3:**
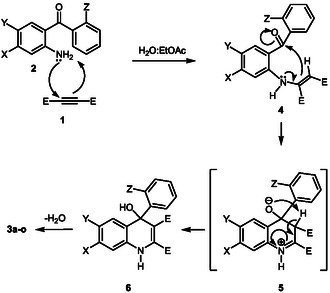
Proposed mechanism for the synthesis of products **3a–o**.

These findings contribute to a deeper understanding of the reaction mechanism and offer valuable insights for further optimization and expansion of synthetic methodologies. The demonstrated versatility and tunability of the reaction enable the targeted synthesis of 4‐aryl quinoline derivatives with specific substituents and high yields. A plausible mechanism for the formation of products **3a–o** is outlined in **Scheme** **3**. Initially, the nucleophilic amine attacks the acetylenic ester, generating the transient intermediate **4**. This is followed by an internal nucleophilic attack, resulting in the formation of intermediate 5, which subsequently undergoes a proton transfer to yield intermediate **6**. Finally, dehydration affords the desired quinoline product **3**.

## 3 Conclusions

We have developed a straightforward, efficient, and environmentally sustainable method for the synthesis of quinolines. This protocol employs a water–ethyl acetate (20:80) solvent system under reflux conditions, eliminating the need for expensive catalysts or hazardous reagents. The resulting products were obtained in high‐purity and excellent yields through simple crystallization from diethyl ether. Key advantages of this methodology include its operational simplicity, the complete avoidance of metal catalysts and inert atmospheres, the use of water as a green solvent, and significantly reduced reaction times. These features make it a desirable and scalable approach for the synthesis of quinolines.

Moreover, the structural similarity between the synthesized quinolines and 8‐hydroxyquinolines underscores their potential applications in organic electronic devices. Future research could explore their ability to form metal complexes, opening new avenues in organic electronics and advanced materials science.

## 4 Experimental Section

4.1

4.1.1

##### General Instrumentation

All purchased solvents and chemicals were of analytical grade and used without further purification. Melting points and IR spectra were measured on an Electrothermal 9100 apparatus and a Shimadzu IR‐470 spectrometer, respectively. Elemental analyses were performed using a Heraeus CHNO‐Rapid analyzer. ^1^H, ^13^C‐NMR spectra were recorded on DRX‐400AVANCE spectrometer at 500.13, 300.13, and 125.77, 75.50 MHz. The results for each product were compared with previous data from our earlier work, which confirmed the structures.

##### General Procedure for the Synthesis of Quinolines 3a–o

A mixture of acetylenic ester (2 mmol) and 2‐amino benzophenone derivatives (2 mmol) was refluxed in a 20:80 (v/v) mixture of H2O and EtOAc for 1 h. The reaction progress was monitored by (TLC) thin layer chromatography (EtOAc:n‐hexane). After the reaction was completed, some crystals formed, even in a warm solution. The reaction medium was then allowed to cool to room temperature, and the precipitate was filtered off. The filtrate was dissolved in warm diethyl ether and allowed to cool in the refrigerator. After a few hours, the recrystallized product could be filtered off. All known compounds have been compared with authentic samples, and the purity of all compounds was confirmed through cross‐melting point analysis.

*Dimethyl 6‐chloro‐4‐phenyl quinoline‐2,3‐dicarboxylate (3a):*

White powder; yield: 89%, mp 158–160 °C.^[^ ^31^ ^]^

IR (KBr): C_*sp*2_—H 3074, C_*sp*3_—H 2958, C=O 1732, 1729 cm^−1^.

^1^H NMR (300.13 MHz, CDCl_3_) : *δ*_H_ 8.29 (d, ^3^ *J*_H‐H_ = 9 Hz, 1H, ‐Ph), 7.79 (dd, ^3^ *J*_H‐H_ = 9 Hz, ^4^ *J*_H‐H_ = 2.3 Hz, 1H, ‐Ph), 7.62 (d, ^4^ *J*_H‐H_ = 2.3 Hz, 1H, ‐Ph), 7.50–7.53 (m, 3H, ‐Ph), 7.36–7.39 (m, 2H, ‐Ph), 4.09 (s, 3H, OCH_3_), 3.66 (s, 3H, OCH_3_).

^13^C NMR (75.5 MHz, CDCl_3_): *δ*_C_ 167.3 and 165.2 (2C=O), 147.2, 145.4, 144.9, 135.6, 133.7, 132.2, 132.1, 129.2, 129.1, 128.5, 128.4, 128.0 and 125.4 (aromatic carbons), 53.5, and 52.6 (2OCH_3_).

MS, m/z (%): (mw 355.78) 355 (30, M+), 357 (10), 326 (6), 324 (16), 298 (10), 296 (31), 241 (34), 239 (100), 204 (30), 176 (18), and 77 (5).

*Dimethyl 4‐phenyl quinoline‐2,3‐dicarboxylate (3b):*

White powder; yield: 92%, mp 123–125 °C.^[^ ^31^ ^]^

IR (KBr): C_*sp*2_–H 3050, C_*sp*3_–H 2995, C=O 1725 cm^−1^.

^1^H NMR(500.13 MHz, CDCl_3_): *δ*_H_ 8.3 *(*d, ^3^ *J*_H‐H_ = 8.5 Hz, 1H, CH), 7.85 (ddd, ^3^ *J*_H‐H_ = 8.5 Hz, ^3^ *J*_H‐H_ = 7.7 Hz, ^4^ *J*_H‐H_ = 1.4 Hz, 1H, CH), 7.62 (d, ^3^ *J*_H‐H_ = 7.7 Hz, 1H, CH), 7.53 (t, ^3^ *J*_H‐H_ = 7.7 Hz, 1H, CH), 7.48 (t, ^3^ *J*_H‐H_ = 6.4 Hz, 1H, CH), 7.52 (d, ^3^ *J*_H‐H_ = 2.0 Hz, 2H, 2CH), 7.39 (dd, ^3^ *J*_H‐H_ = 6.4 Hz, ^3^ *J*_H‐H_ = 2.0 Hz, 2H, 2CH), 4.10 (s, 3H, OCH_3_), and 3.66 (s, 3H, OCH_3_).

^13^C NMR (125.77 MHz, CDCl_3_): *δ*_C_ 167.5 and 165.5 (2C=O), 148.0, 147.1, 144.82, 134.5, 131.08, 130.6, 129.3, 129.1, 128.8, 128.2, 127.6, 127.1 and 126.6 (aromatic carbons), 53.54, and 52.51 (2OCH_3_).

MS, m/z (%): (mw 321.34) 321 (11, M+), 262 (21), 204 (100), 203 (44), 77 (8), and 59 (81).

*Dimethyl 6‐chloro‐4‐(2‐chlorophenyl)quinoline‐2,3‐dicarboxylate (3c)*:

Yellow crystals; yield: 81%, mp 191–193 °C.^[^ ^31^ ^]^

IR (KBr): C_*sp*2_–H 3070, C_*sp*3_–H 2985, C=O 1729, 1720 cm^−1^.

^1^H NMR (500.13 MHz, CDCl_3_): *δ*_H_ 8.27 (d, 3 *J*_HH_ = 9 Hz, 1H, ‐Ph), 7.77 (dd, 3 *J*_HH_ = 9 Hz, 4 *J*_HH_ = 2.1 Hz, 1H, ‐Ph), 7.57 (d, 3 *J*_HH_ = 8 Hz, 1H, ‐Ph), 7.48 (t, 3 *J*_HH_ = 7.5 Hz, 1H, ‐Ph), 7.42 (t, 3 *J*_HH_ = 7.5 Hz, 1H, ‐Ph), 7.36 (d, 4 *J*_HH_ = 2 Hz, 1H, ‐Ph), 7.28 (d, 3 *J*_HH_ = 7.4 Hz, 1H, ‐Ph), 4.06 (s, 3H, OCH_3_), and 3.63 (s, 3H, OCH_3_).

^13^C NMR (125.77 MHz, CDCl3): *δ*_C_ 166.6, 165.3 (2C=O), 145.9, 145.4, 144.9, 135.9, 133.5, 132.9, 132.4, 132.2, 130.9, 130.7, 129.8, 127.9, 127.7, 126.8 and 124.9 (aromatic carbons), 53.5 (OCH_3_), and 52.6 (OCH_3_).

*Dimethyl 6‐chloro‐4‐(2‐fluorophenyl)quinoline‐2,3‐dicarboxylate (3d):*

White crystals; yield: 94%, mp 209–212 °C.^[^ ^31^ ^]^

IR (KBr): C_*sp*2_–H 3079, C_*sp*3_–H 2953, C=O 1729, 1718 cm^−1^.

^1^H NMR (300.13 MHz, CDCl_3_): *δ*_H_ 8.27 (d, 3 *J*_HH_ = 9 Hz, 1H, ‐Ph), 7.71 (dd, 3 *J*_HH_ = 9 Hz,4 *J*_HH_ = 2.1 Hz, 1H), 7.52–7.55 (m, 1H, ‐Ph), 7.49 (s, 1H, ‐Ph), 7.24–7.33 (m, 3H, ‐Ph), 4.06 (s, 3H, OCH3), and 3.66 (s, 3H, OCH_3_).

^13^C NMR (75.5 MHz, CDCl_3_): *δ*_C_ 166.7 and 165.2 (2C=O), 159.5 (d, 1 *J*_FC_ = 249.3 Hz), 145.7, 145.3, 141.8, 135.9, 132.4, 132.2, 131.6 (d, 3 *J*_FC_ = 8.1 Hz), 131.1 (d, 3 *J*_FC_ = 2.4 Hz), 128.4, 128.2, 124.9, 124.3 (d, 2 *J*_FC_ = 3.5 Hz), 121.5 (d, 2 *J*_FC_ = 16.8 Hz), 115.9 (d, 2 *J*F_C_  = 21.1 Hz), (aromatic carbons), 53.5 (OCH_3_), and 52.6 (OCH_3_).

*Dimethyl 7‐methyl‐4‐phenylquinoline‐2,3‐dicarboxylate (3e):*

White powder; yield: 89%, mp 158–160 °C.

IR (KBr): C_*sp*2_–H 3074, C_*sp*3_–H 2958, C=O 1732, 1729 cm^−1^.

^1^H NMR (300.13 MHz, CDCl_3_): *δ*_H_ 8.29 (d, ^3^ *J*_H‐H_ = 9 Hz, 1H, ‐Ph), 7.79 (dd, ^3^ *J*_H‐H_ = 9 Hz, ^4^ *J*_H‐H_ = 2.3 Hz, 1H, ‐Ph), 7.62 (d, ^4^ *J*_H‐H_ = 2.3 Hz, 1H, ‐Ph), 7.50–7.53 (m, 3H, ‐Ph), 7.36–7.39 (m, 2H, ‐Ph), 4.09 (s, 3H, OCH3), and 3.66 (s, 3H, OCH_3_).

^13^C NMR (75.5 MHz, CDCl_3_): *δ*_C_ 167.2 and 165.2 (2C=O), 147.2, 145.4, 144.9, 35.6, 133.7, 132.1, 131.9, 129.2, 129.1, 128.5, 128.4, 128.0 and 125.4 (aromatic carbons), 53.5, and 52.6 (2OCH_3_).

*Diethyl 6‐chloro‐4‐phenylquinoline‐2,3‐dicarboxylate (3f):*

White powder; yield: 93%, mp 165–167 °C.^[^ ^31^ ^]^

IR (KBr): C_*sp*2_–H 3075, C_*sp*3_–H 2988, C=O 1739, 1724 cm^−1^.

^1^H NMR (300.13 MHz, CDCl_3_) : *δ*_H_ 8.26 (d, ^3^ *J*_H‐H_ = 8.26 Hz, 1H, ‐Ph), 7.74 (dd, ^3^ *J*_H‐H_ = 9 Hz, ^4^ *J*_H‐H_ = 2.1 Hz,1H, ‐Ph), 7.57 (d, ^4^ *J*_H‐H_ = 2.1 Hz, 1H, ‐Ph), 7.50–7.53 (m, 3H, ‐Ph), 7.33–7.36 (m, 2H, ‐Ph), 4.52 (q, ^3^ *J*_H‐H_ = 7.1 Hz, 2H, OCH_2_), 4.09 (q, ^3^ *J*_H‐H_ = 7.1 Hz, 2H, OCH_2_), 1.45 (t, ^3^ *J*_H‐H_ = 7.1 Hz, 3H, CH_3_), and 0.98 (t, ^3^ *J*_H‐H_ = 7.1 Hz, 3H, CH_3_).

^13^C NMR (75.5 MHz, CDCl_3_): *δ*_C_ 166.7, 164.9 (2C=O), 147.1, 145.9, 145.4, 135.3, 134.0, 132.2, 131.9, 129.3, 129.0, 128.4, 128.3, 127.9 and 125.3 (aromatic carbons), 62.7 (OCH_2_), 61.6 (OCH_2_), 14.1 (CH_3_), and 13.5 (CH_3_).

*Diethyl 4‐phenylquinoline‐2,3‐dicarboxylate (3g):*

White crystals; yield: 92%, mp 96–99 °C.^[^ ^31^ ^]^

IR (KBr): C_*sp*2_–H 3030, C_*sp*3_–H 2985, C=O 1728 cm^−1^.

^1^H NMR (500.13 MHz, CDCl_3_) : *δ*_H_ 8.29 (d, 3 *J*_HH_ = 8.5 Hz, 1H, CH), 7.76 (ddd, 3 *J*_HH_ = 8.5 Hz, 3 *J*_HH_ = 7.8 Hz, 4 *J*_HH_ = 1.4 Hz, 1H, CH), 7.59 (d, 3 *J*_HH_ = 7.8 Hz, 1H, CH), 7.53 (t, 3 *J*_HH_ = 7.8 Hz, 1H, CH), 7.46 (t, 3 *J*_HH_ = 6.5 Hz, 1H, CH), 7.45 (d, 3 *J*_HH_ = 2.2 Hz, 2H, 2CH), 7.33 (dd, 3 *J*_HH_ = 6.5 Hz, 3 *J*_HH_ = 2.2 Hz, 2H, 2CH), 4.50 (q, 3 *J*_HH_ = 7.1 Hz, 2H, OCH_2_), 4.06 (q, 3 *J*_HH_ = 7.1 Hz, 2H, OCH_2_), 1.43 (t, 3 *J*_HH_ = 7.1 Hz, 3H, CH_3_), and 0.96 (t, 3 *J*_HH_ = 7.1 Hz, 3H, CH_3_).

^13^C NMR (125.77 MHz, CDCl_3_): *δ*_C_ 167.0 and 165.2 (2C=O), 147.9, 147.1, 146.0, 134.8, 130.8, 130.6, 129.9, 128.9, 128.7, 128.2, 127.5, 127.0 and 126.5 (aromatic carbons), 62.5 and 61.4 (2OCH_2_), 14.1, and 13.5 (2CH_3_).

*Diethyl 6‐chloro‐4‐(2‐chlorophenyl)quinoline‐2,3‐dicarboxylate (3h):*

White crystals; yield: 89%, mp 126–129 °C.^[^ ^31^ ^]^

IR (KBr): C_*sp*2_–H 3074, C_*sp*3_–H 2983, C=O 1729, 1724 cm^−1^.

^1^H NMR (300.13 MHz, CDCl_3_) : *δ*_H_ 8.27 (d, 3 *J*_HH_ = 9 Hz, 1H, ‐Ph), 7.76 (d, 3 *J*_HH_ = 9 Hz, 1H, ‐Ph), 7.56 (d, 3 *J*_HH_ = 9 Hz, 1H, ‐Ph), 7.48 (t, 3 *J*_HH_ = 7.4 Hz, 1H, ‐Ph), 7.41 (t, 3 *J*_HH_ = 7.4 Hz, 1H, ‐Ph), 7.36 (s, 1H, ‐Ph), 7.28 (d, 3 *J*_HH_ = 7.6 Hz, 1H, ‐Ph), 4.52 (q, 3 *J*_HH_ = 7.1 Hz, 2H, OCH_2_), 4.06–4.15 (m, 2H, OCH_2_), 1.45 (t, 3 *J*_HH_ = 7.1 Hz, 3H, CH3), and 1.00 (t, 3 *J*_HH_ = 7.1 Hz, 3H, CH_3_).

^13^C NMR (75.5 MHz, CDCl_3_): *δ*_C_ 166.0, 164.9 (2C=O), 146.8, 145.4, 144.7, 135.6, 133.6, 133.2, 132.3, 132.2, 131.1, 130.6, 129.7, 127.8, 127.6, 126.7 and 124.9 (aromatic carbons), 62.6 (OCH_2_), 61.7 (OCH_2_), 14.1 (CH_3_), and 13.5 (CH_3_).

*Diethyl 6‐chloro‐4‐(2‐fluorophenyl)quinoline‐2,3‐dicarboxylate (3i):*

White crystals; yield: 95%, mp 136–138 °C.^[^ ^31^ ^]^

IR (KBr): C_*sp*2_–H 3084, C_*sp*3_–H 2983, C=O 1729, 1719 cm^−1^.

^1^H NMR (300.13 MHz, CDCl3): *δ*_H_ 8.25 (d, 3 *J*_HH_ = 9 Hz, 1H, ‐Ph), 7.74 (dd, 3 *J*_HH_ = 9 Hz, 4 *J*_HH_ = 2.3 Hz, 1H, Ph), 7.50–7.53 (m, 2H, ‐Ph), 7.49 (s, 1H, ‐Ph), 7.22–7.29 (m, 3H, ‐Ph), 4.51 (q, 3 *J*_HH_ = 7.1 Hz, 2H, OCH_2_), 4.08–4.13 (m, 2H, OCH_2_), 1.44 (t, 3 *J*_HH_ = 7.1 Hz, 3H, CH_3_), and 1.01 (t, 3 *J*_HH_ = 7.1 Hz, 3H, CH_3_).

^13^C NMR (75.5 MHz, CDCl_3_): *δ*_C_ 166.2, 164.9 (2C=O), 159.6 (d, 1 *J*_FC_ = 248.8 Hz), 146.6, 145.3, 141.6, 135.6, 131.4 (d, 3 *J*_FC_ = 7.8 Hz), 131.2 (d, 3 *J*_FC_ = 2.5 Hz), 132.2, 132.1, 128.3, 128.1, 124.9, 124.2 (d, 3 *J*_FC_ = 3.4 Hz), 121.8 (d, 2 *J*_FC_ = 16.9 Hz) and 115.9 (d, 2 *J*_FC_ = 21.2 Hz) (aromatic carbons), 62.6 (OCH_2_), 61.7 (OCH_2_), 14.1, and 13.5 (2CH_3_).

*Diethyl 7‐methyl‐4‐phenylquinoline‐2,3‐dicarboxylate (3j):*

Yellow crystals; yield: 91%, mp 152–155 °C.

*Di‐tert‐butyl 6‐chloro‐4‐(2‐chlorophenyl)quinoline‐2,3‐dicarboxylate (3k):*

Yellow crystals; yield: 77%, mp 227–230 °C.

*Di‐tert‐butyl 6‐chloro‐4‐(2‐fluorophenyl)quinoline‐2,3‐dicarboxylate (3l):*

Yellow crystals; yield: 85%, mp 124–126 °C.

*Di‐tert‐butyl 7‐methyl‐4‐phenylquinoline‐2,3‐dicarboxylate (3m):*

Yellow crystals; yield: 79%, mp 141–143 °C.

*Di‐tert‐butyl 4‐phenylquinoline‐2,3‐dicarboxylate (3n):*

White crystals; yield: 92%, mp 107–109 °C.

*Di‐tert‐butyl 6‐chloro‐4‐phenylquinoline‐2,3‐dicarboxylate (3o):*

White crystals; yield: 90%, mp 138–141 °C.

## Conflict of Interest

The authors declare no conflict of interest.

## Supporting information

Supplementary Material

## Data Availability

The data that support the findings of this study are available in Supporting Information of this article.
