# Supplementary material for: Exploring a Water–Ethyl Acetate System for the Efficient Synthesis of 4‐Aryl Quinolines
Source: ChemistryOpen. 2025 Apr 7;14(8):e202400470. doi: 10.1002/open.202400470 (PMC12368872; doi:10.1002/open.202400470)
Supplement: Supplementary file 1 — Supplementary Material [file OPEN-14-e202400470-s001.pdf]

Exploring a Water-Ethyl Acetate System for the Efficient Synthesis of 4-Aryl Quinolines

Mohammad Qandalee,*,[a] Aliyeh Khajeh-Khezri,[b] Mohammad Alikarami,[c] Carlos J. Durán-Valle,[d] Silvia Izquierdo,[e] Ignacio M. López-Coca.*,[e]

[a] *Department of Basic Sciences, Garmsar Branch, Islamic Azad University, Garmsar, Iran.*

[b] *Department of Chemistry, Tarbiat Modares University, Tehran, Iran*

[c] *Department of Chemistry, Ilam Branch, Islamic Azad University, Ilam, Iran*

[d] *IACYS, Faculty of Sciences, University of Extremadura, Badajoz - 06006, Spain*

[e] *INTERRA, School of Technology, University of Extremadura, Cáceres - 10003, Spain*

** E-mail: qandalee@gmail.com, iglomar@unex.es*

SUPPLEMENTARY INFORMATION

General procedure for the synthesis of quinolones

A mixture of acetylenic ester (2 mmol) and 2-amino benzophenone derivatives (2 mmol) was refluxed in H_2O: EtOAc (2:8 ml) for 1 h. The reaction progress was monitored by TLC. After completion of the reaction, the precipitate was filtered and purified by recrystallization from diethyl ether. The products were compared with authentic samples.

Dimethyl 6-chloro-4-phenyl-2,3-quinolinedicarboxylate (3a)

White powder; yield: 89%, mp 158–160 ◦C. IR (KBr): C*sp*2– H 3074, C*sp*3–H 2958, C=O 1732, 1729 cm−.

^{1}H NMR (300.13, CDCl3) : *δ*H 8.29 (d, 3 *J*HH = 9Hz,1H, -Ph), 7.79 (dd, 3 *J*HH = 9Hz,4 *J*HH = 2.3Hz, 1H,-Ph), 7.62 (d, 4 *J*HH = 2.3Hz, 1H, -Ph), 7.50-7.53 (m, 3H, -Ph), 7.36-7.39 (m, 2H, -Ph), 4.09 (s,3H,OCH3), 3.66 (s, 3H, OCH3). ^{13}C NMR (75.5MHz, CDCl3): *δ*C 167.3 and 165.2 (2C=O), 147.2, 145.4, 144.9, 135.6, 133.7, 132.2, 132.1, 129.2, 129.1, 128.5, 128.4, 128.0 and 125.4 (aromatic carbons), 53.5, 52.6 (2OCH_3).

MS,m/z (%): 321 (11, M+), 262 (21), 204 (100), 203 (44), 77 (8), 59 (81).

Anal. Cacld. for $C_{19}H_{15}NO_4$ (321.34): C 71.02, H 4.71, N 4.36%. Found: C 70.60, H 4.89, N 3.63%.

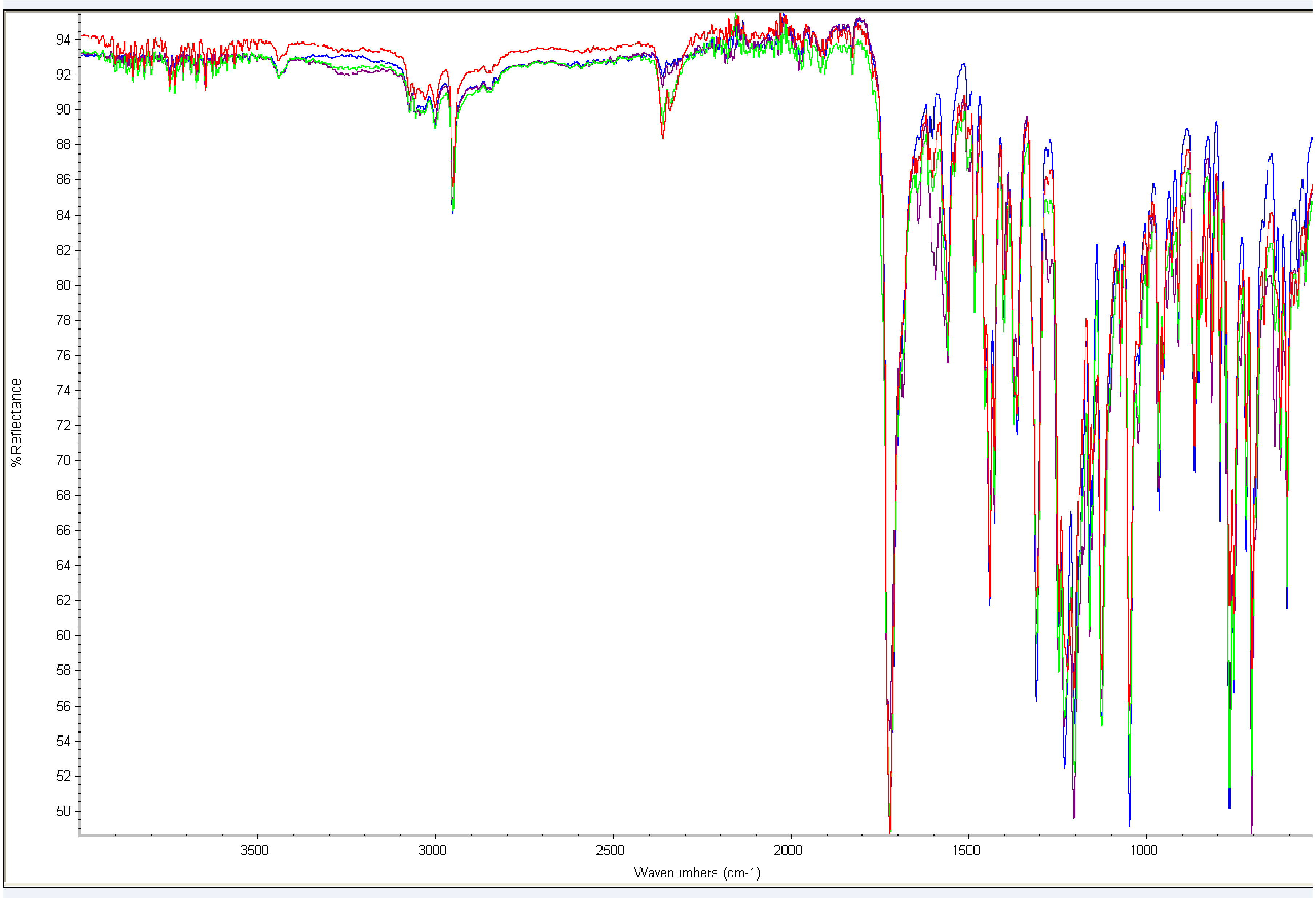

Figure S1: IR spectrum of compound 3a

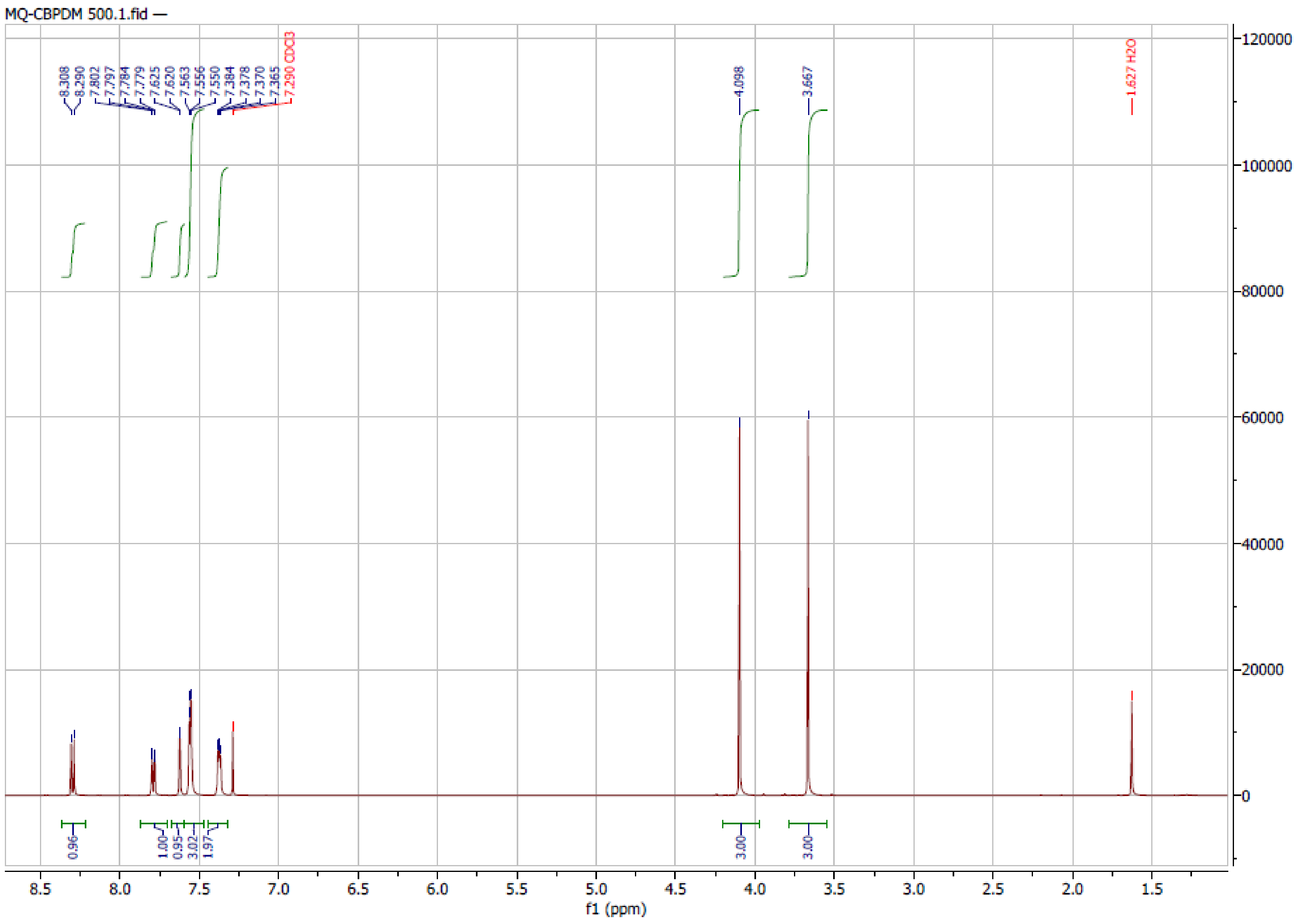

Figure S2: ^{1}HNMR spectrum of compound 3a

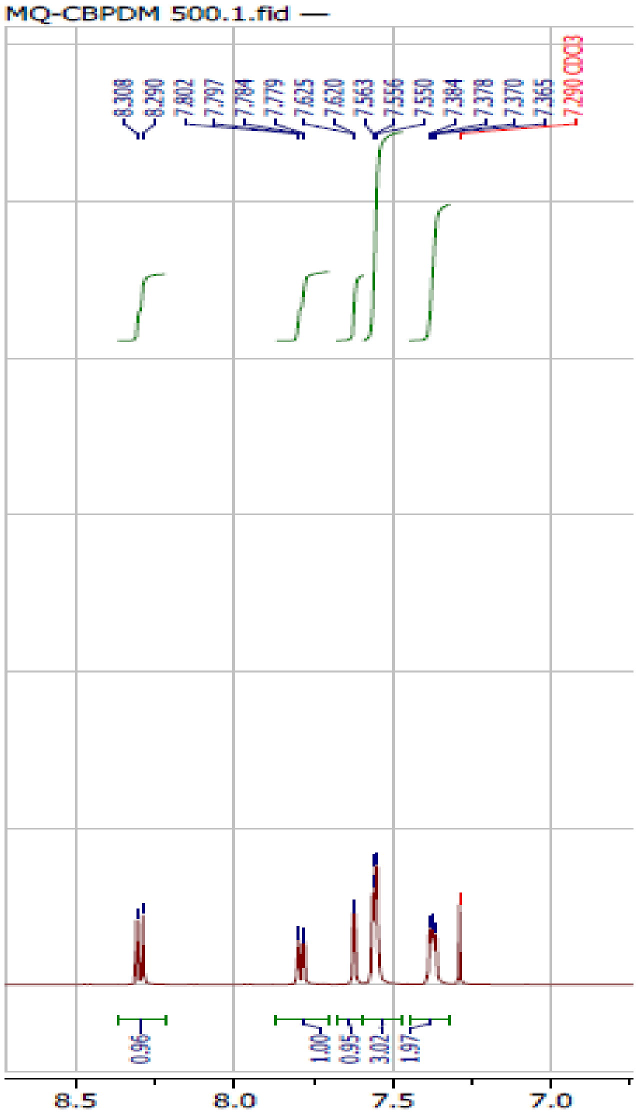

Figure S2 (zoom): ^{1}HNMR spectrum of compound 3a

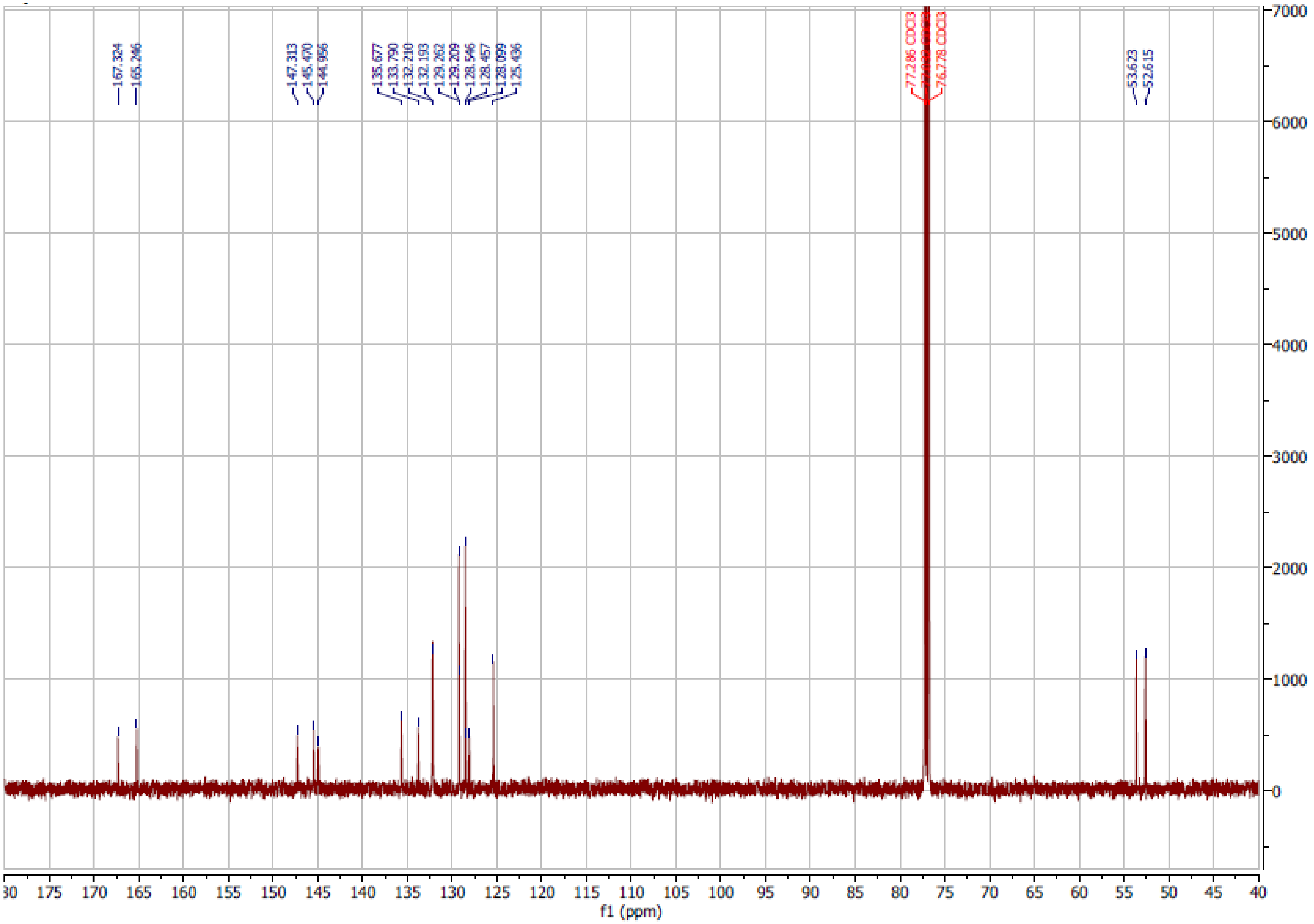

Figure S3: ^{13}CNMR spectrum of compound 3a

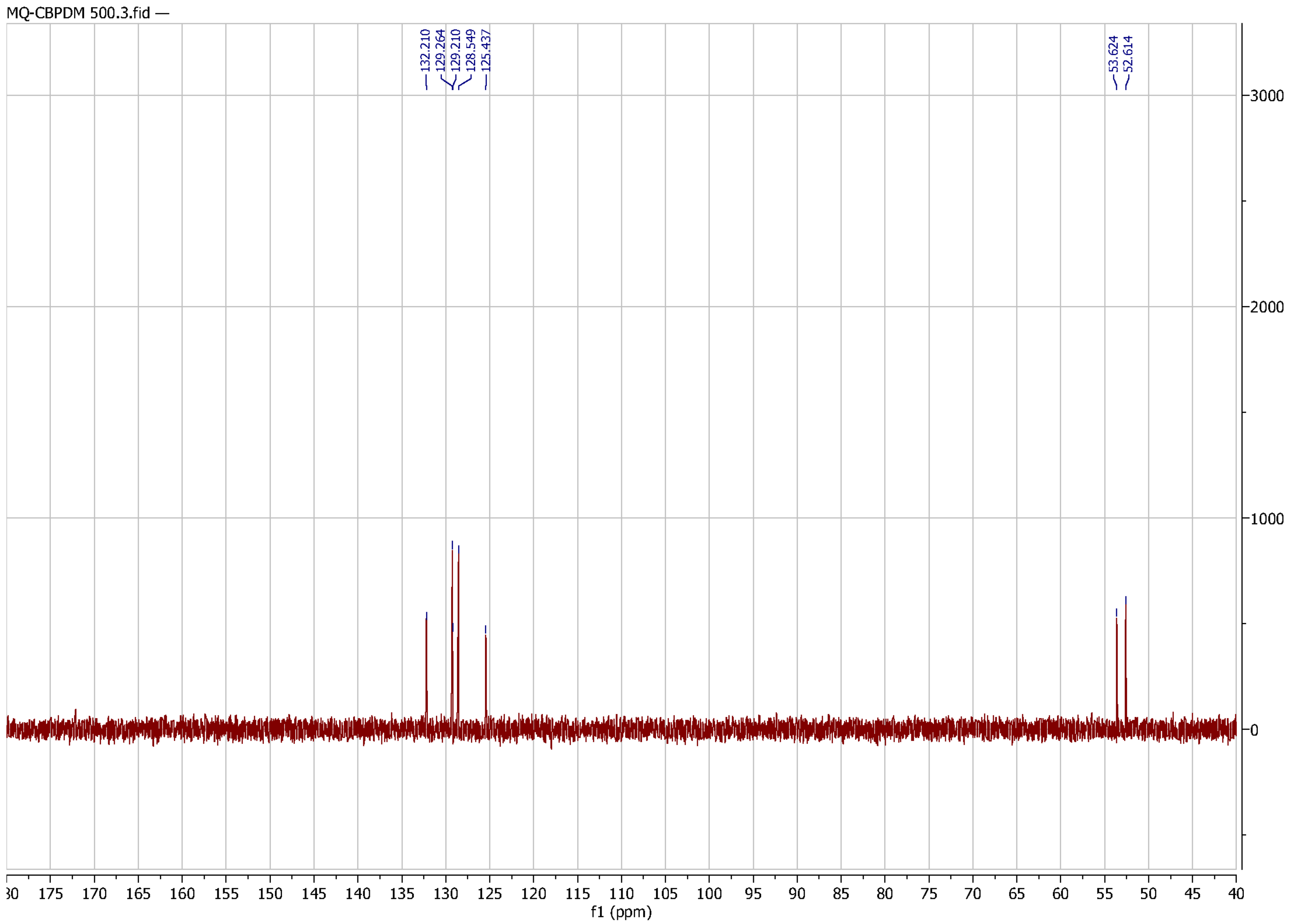

Figure S4: ^{13}CNMR DEPT spectrum of compound 3a

Dimethyl 4-phenyl-2,3-quinolinedicarboxylate (3b)

White powder; yield: 92%, mp 123–125 ◦C; IR (KBr): C*sp*2– H 3050, C*sp*3–H 2995, C=O 1725 cm−1.

^{1}H NMR(500.13MHz, CDCl3): *δ*H 8.3 (d, 3 *J*HH=8.5Hz,1H, CH), 7.85 (ddd, 3 *J*HH = 8.5Hz,3 *J*HH = 7.7Hz,4 *J*HH = 1.4Hz, 1H, CH), 7.62 (d, 3 *J*HH = 7.7Hz, 1H, CH),7.53 (t, 3 *J*HH = 7.7Hz, 1H, CH), 7.48 (t, 3 *J*HH = 6.4Hz,1H, CH), 7.52 (d, 3 *J*HH = 2.0Hz, 2H, 2CH), 7.39 (dd,3 *J*HH = 6.4Hz,3 *J*HH 2.0Hz, 2H, 2CH), 4.10 (s, 3H,OCH3),3.66 (s, 3H, OCH3). ^{13}C NMR (125.77MHz, $CDCl_3$): *δ*C 167.5 and 165.5 (2C=O), 148.0, 147.1, 144.82, 134.5, 131.08, 130.6, 129.3, 129.1, 128.8, 128.2, 127.6, 127.1 and 126.6 (aromatic carbons), 53.54 and 52.51 (2OCH3).

Anal. Cacld. for $C_{19}H_{15}NO_4$ (321.34): C 71.02, H 4.71, N 4.36%. Found: C 70.60, H 4.89, N 3.63%.

MS (Exact mass for $C_{19}H_{15}NO_4$: 321.10): Found $(M+H)^+$ 322.1074.

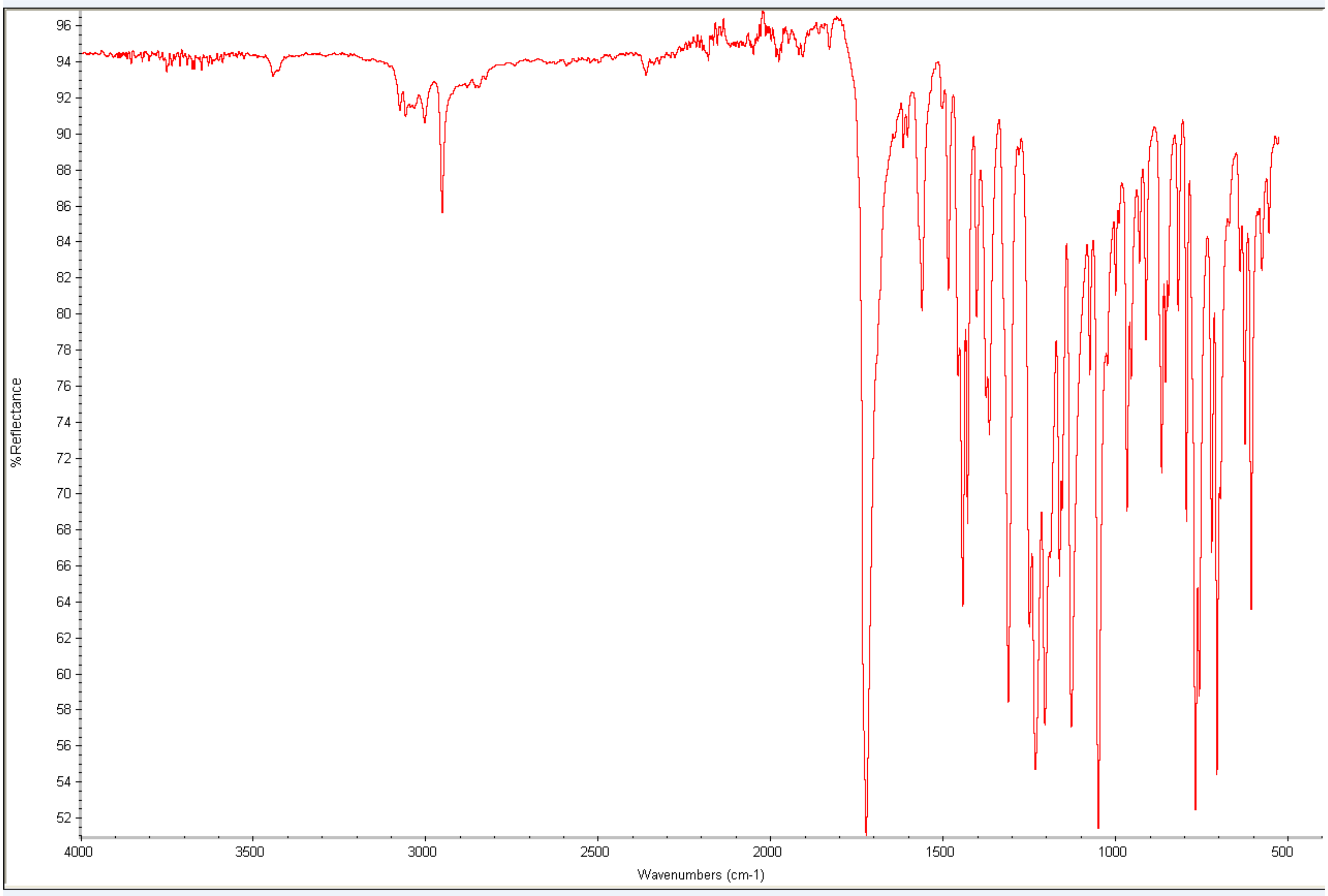

Figure S5: IR spectrum of compound 3b

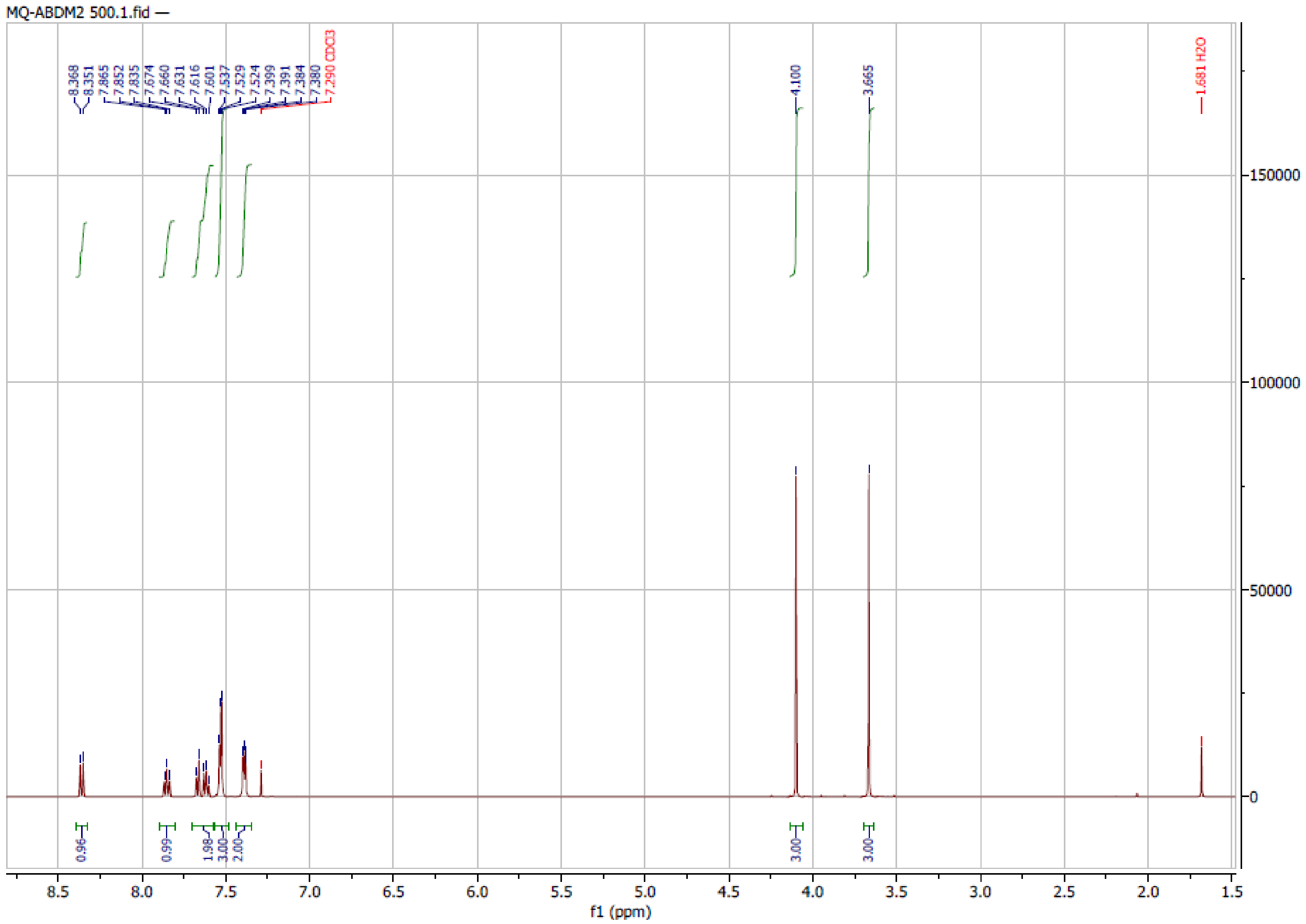

Figure S6: ^{1}HNMR spectrum of compound 3b

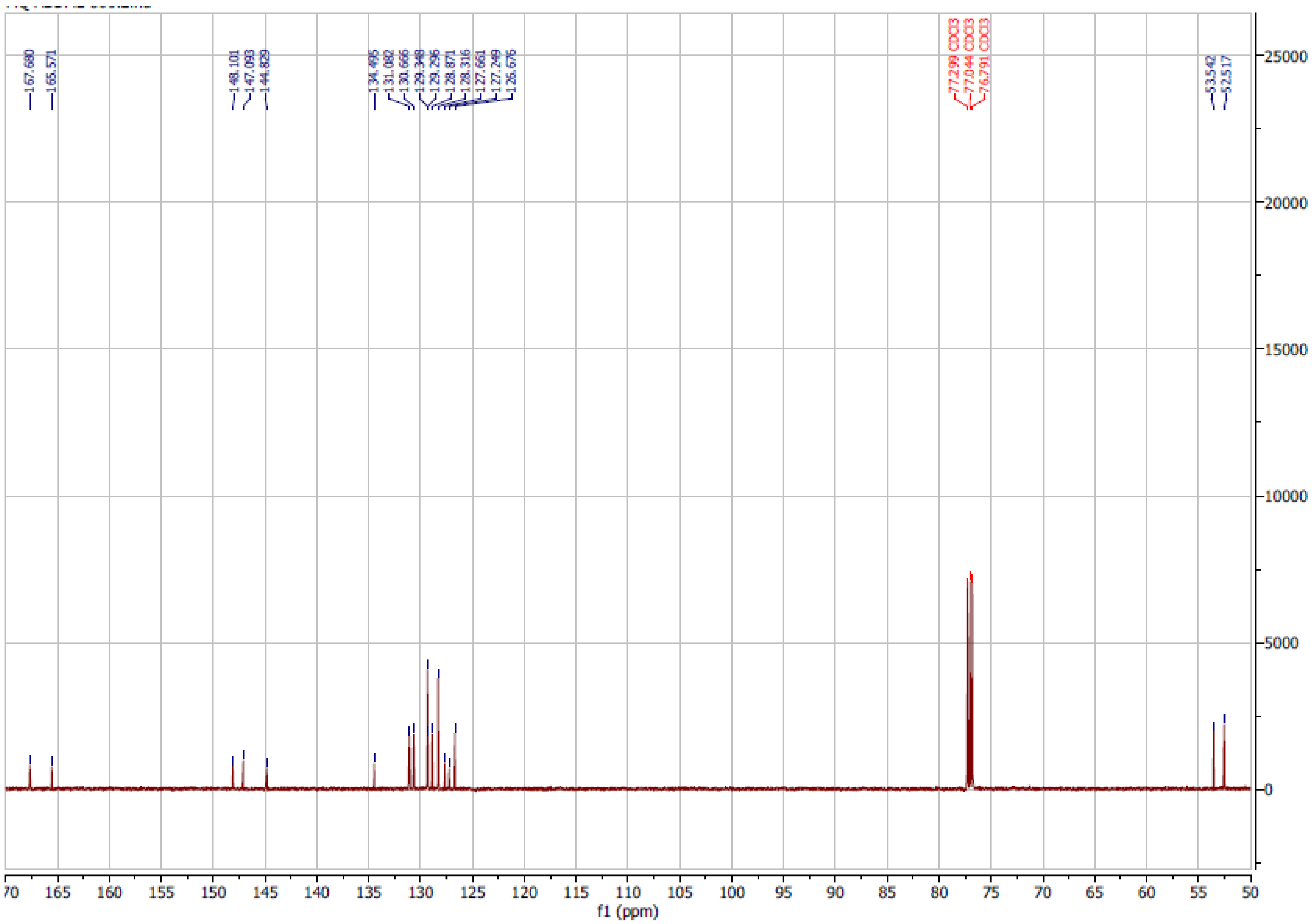

Figure S7: ^{13}CNMR spectrum of compound 3b

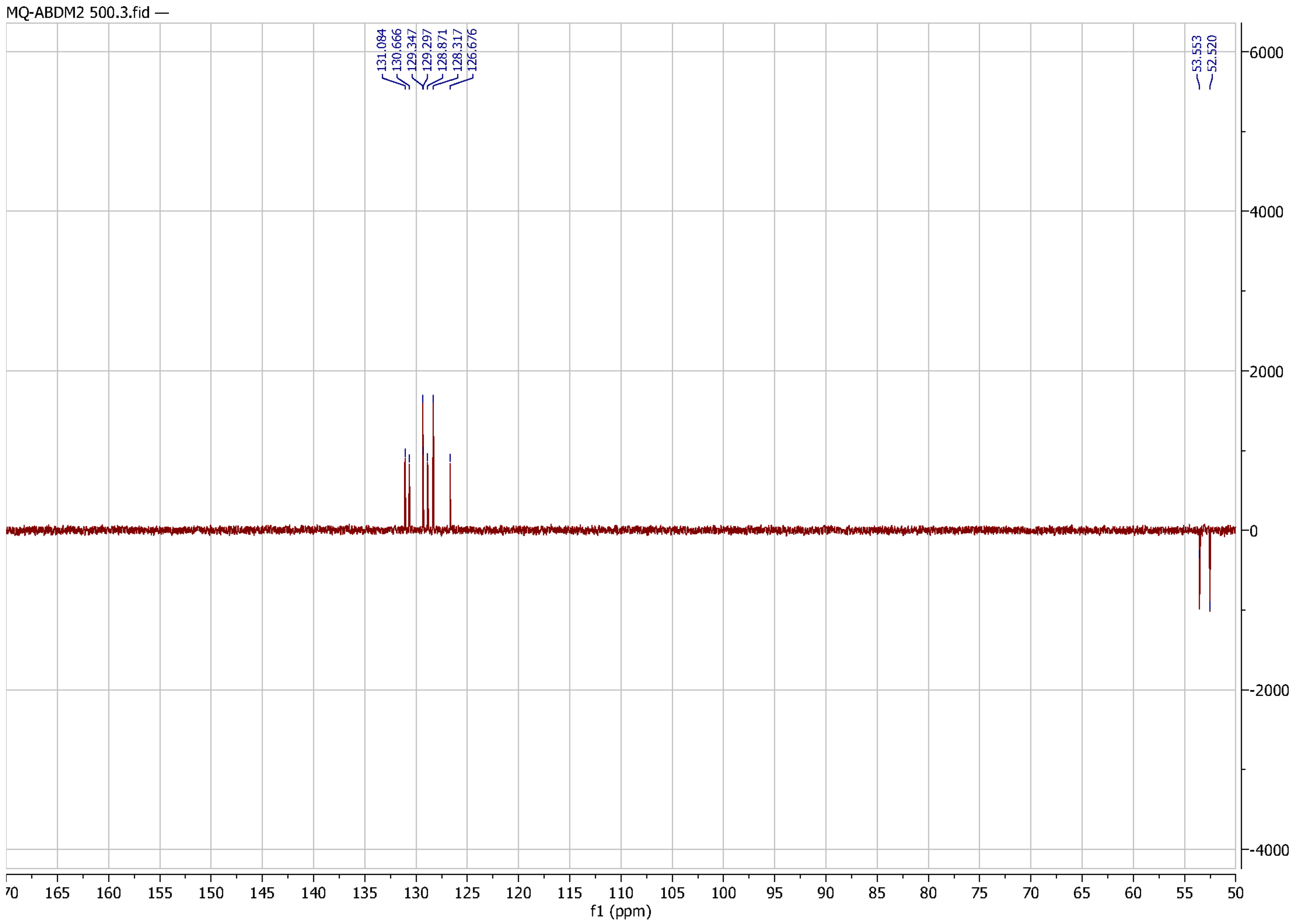

Figure S8: ^{13}CNMR DEPT spectrum of compound 3b

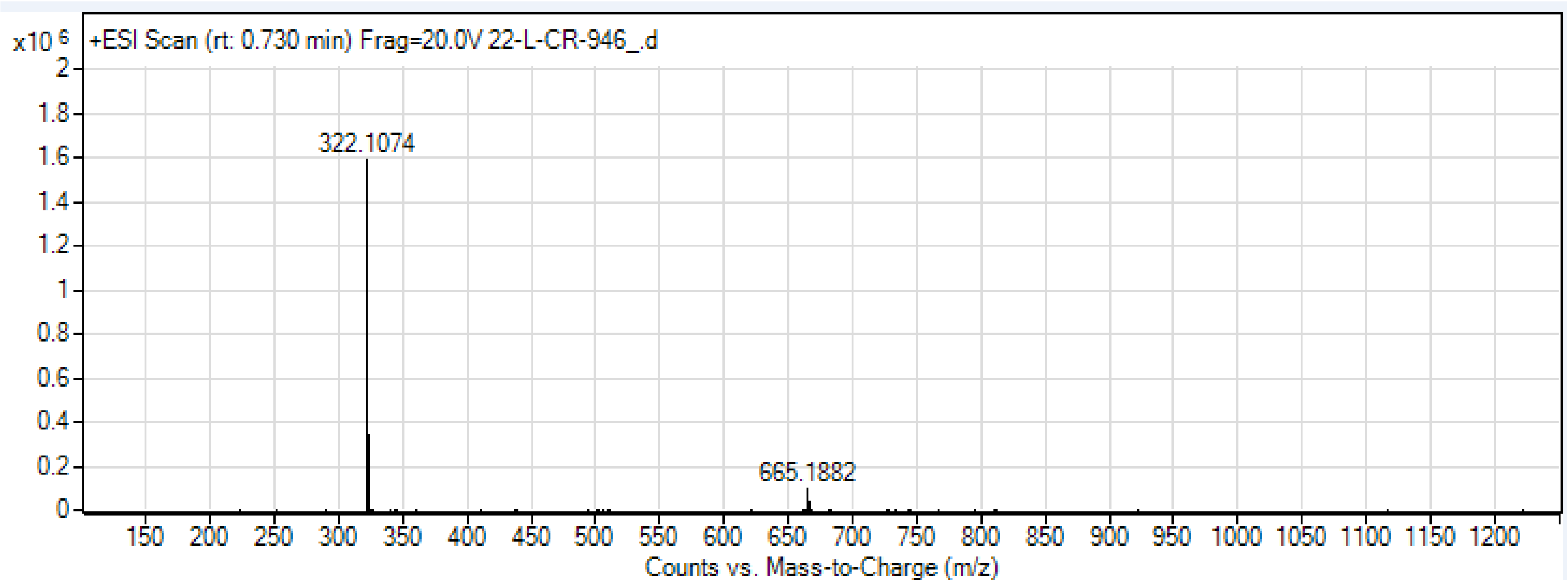

Figure S9: Mass spectrometry of compound 3b

Diethyl 6-chloro-4-phenyl-2,3-quinolinedicarboxylate (3f)

White powder; yield: 93%, mp 165–167 ◦C. IR (KBr): C*sp*2–H 3075, C*sp*3–H 2988, C=O 1739,1724 cm−1.

^{1}H NMR (300.13MHz, CDCl3) : δH 8.26 (d, 3 *J*HH = 8.26 Hz, 1H, -Ph), 7.74 (dd, 3 *J*HH = 9Hz,4 *J*HH = 2.1Hz,1H, -Ph), 7.57 (d, 4 *J*HH = 2.1Hz, 1H, -Ph), 7.50–7.53 (m, 3H, -Ph), 7.33–7.36 (m, 2H, -Ph), 4.52 (q, 3 *J*HH = 7.1Hz, 2H, OCH2), 4.09 (q, 3 *J*HH = 7.1Hz, 2H, OCH2), 1.45 (t, 3 *J*HH = 7.1Hz, 3H, CH3), 0.98 (t, 3 *J*HH = 7.1Hz,3H, CH3). ^{13}C NMR (75.5MHz, CDCl3): δC 166.7, 164.9 (2C=O), 147.1, 145.9, 145.4, 135.3, 134.0, 132.2, 131.9, 129.3, 129.0, 128.4, 128.3, 127.9 and 125.3 (aromatic carbons), 62.7 (OCH2), 61.6 (OCH2), 14.1 (CH3), 13.5 (CH3).

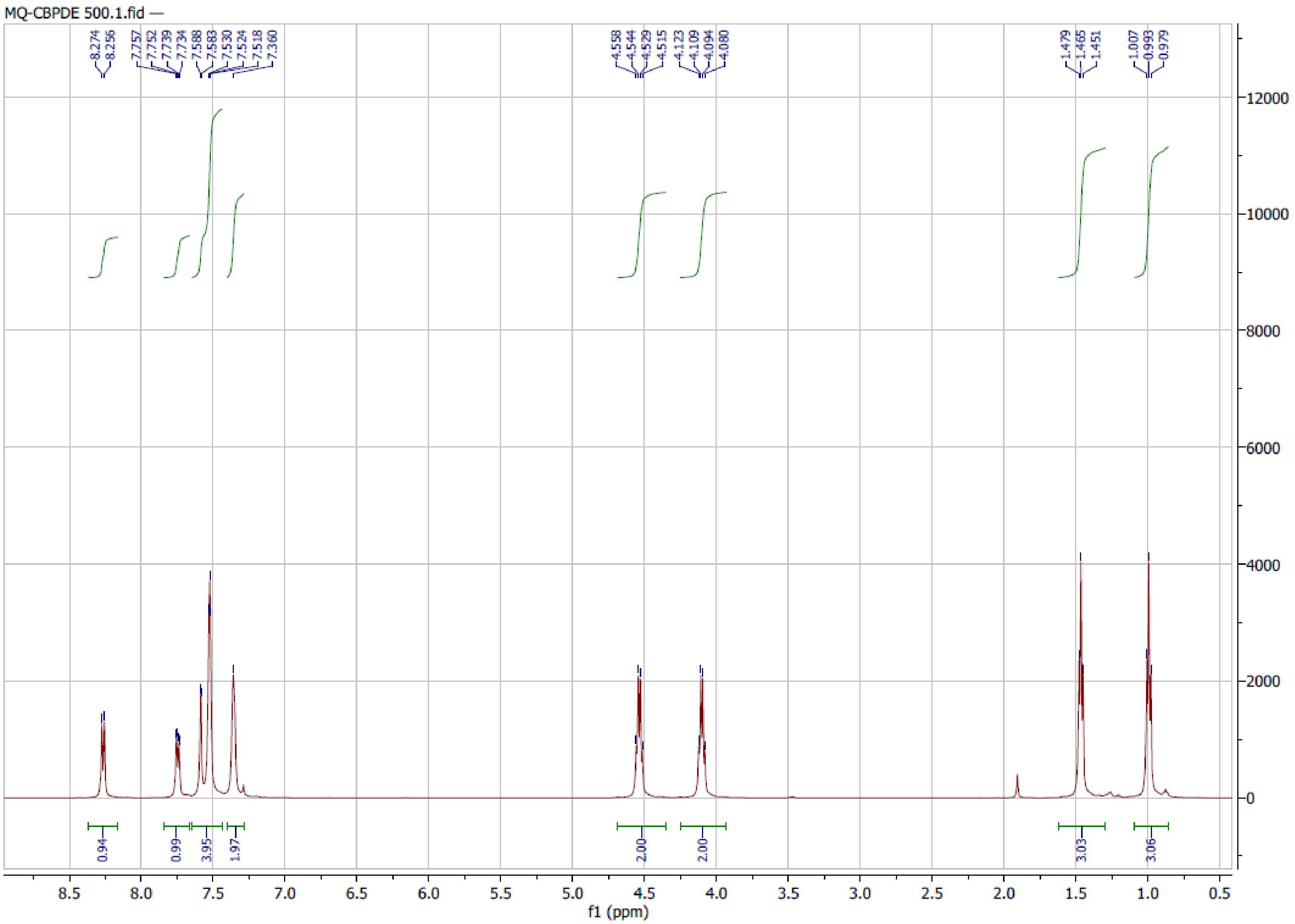

Figure S10: ^{1}HNMR spectrum of compound 3f

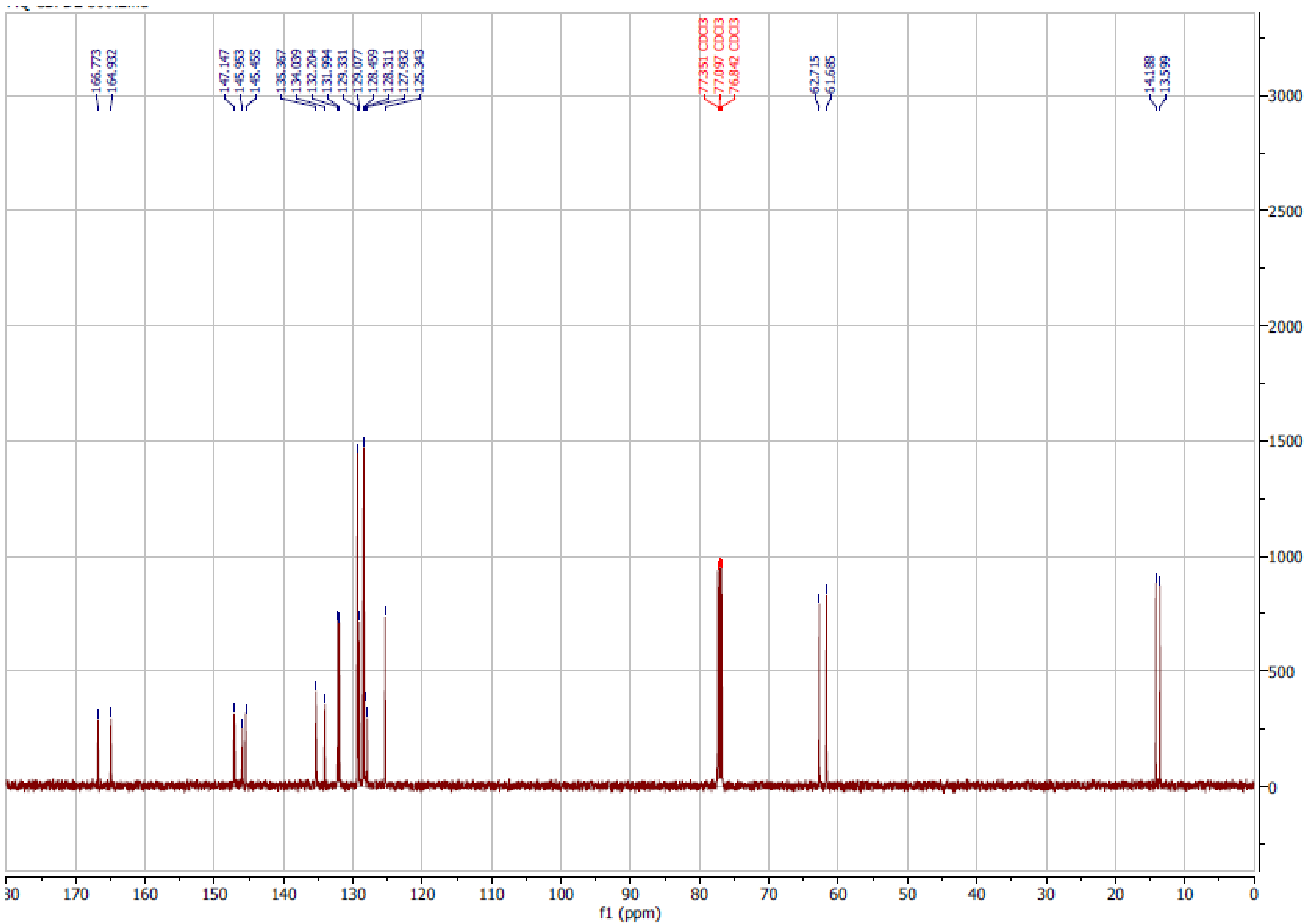

Figure S11: ^{13}CNMR spectrum of compound 3f

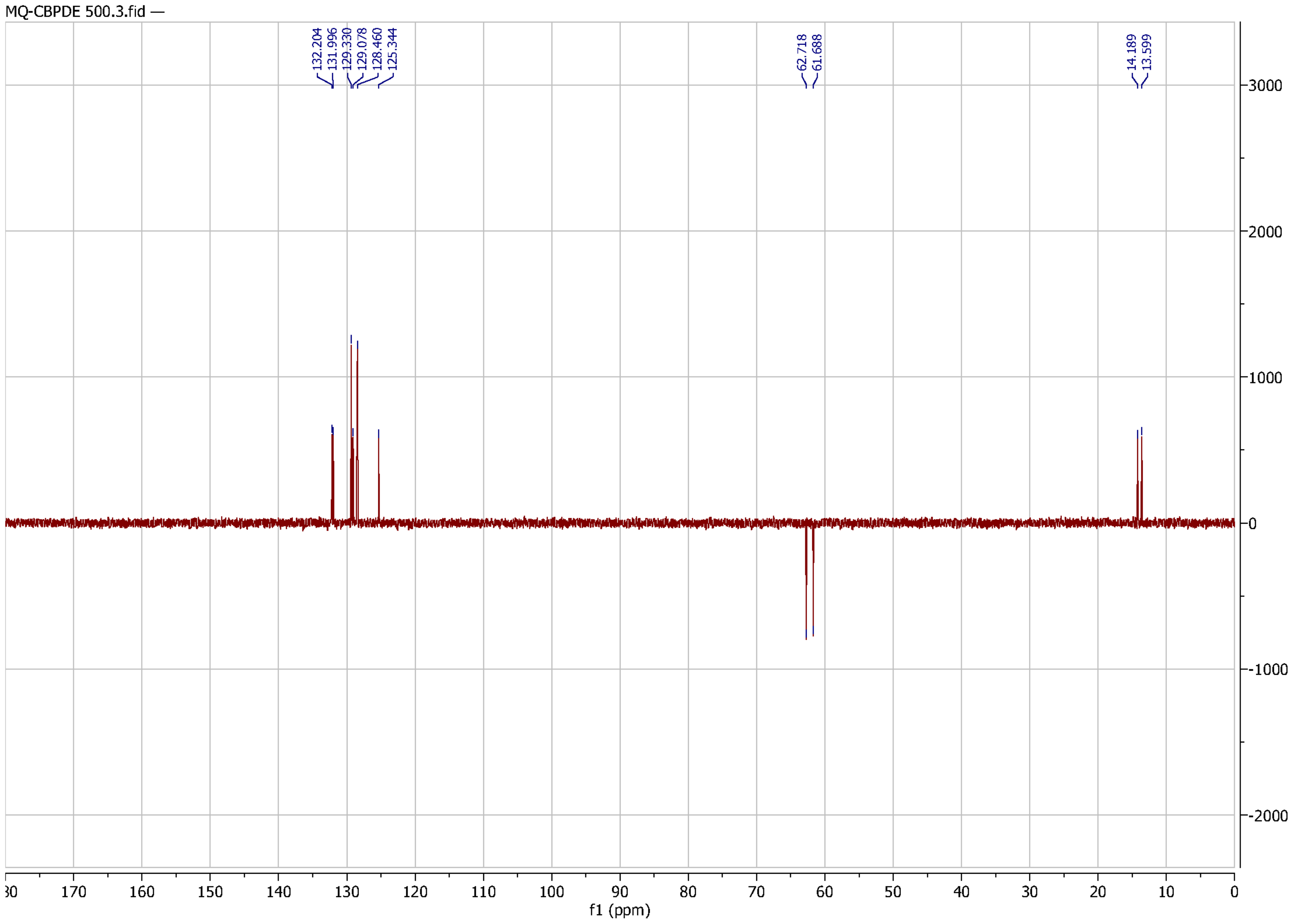

Figure S12: ^{13}CNMR DEPT spectrum of compound 3f

Diethyl 4-phenyl-2,3-quinolinedicarboxylate (3g)

White powder; yield: 93%, mp 95–97 ◦C. IR (KBr): C*sp*2–H 3030, C*sp*3–H 2985, C=O 1728cm−1.

^{1}H NMR (500.13, $CDCl_3$) : *δ*H 8.29 (d, 3 *J*HH = 8.5Hz, 1H, CH), 7.76 (ddd, 3 *J*HH = 8.5Hz,3 *J*HH = 7.8Hz,4 *J*HH = 1.4Hz, 1H, CH), 7.59 (d, 3 *J*HH = 7.8Hz, 1H, CH), 7.53 (t, 3 *J*HH = 7.8Hz, 1H, CH), 7.46 (t, 3 *J*HH = 6.5Hz, 1H, CH), 7.45 (d, 3 *J*HH = 2.2Hz, 2H, 2CH), 7.33 (dd, 3 *J*HH = 6.5Hz,3 *J*HH = 2.2Hz, 2H, 2CH), 4.50 (q, 3 *J*HH =7.1Hz, 2H, OCH2), 4.06 (q, 3 *J*HH = 7.1Hz, 2H, OCH2), 1.43 (t, 3 *J*HH = 7.1Hz, 3H, CH3), 0.96 (t, 3 *J*HH = 7.1Hz, 3H, CH3).

^{13}C NMR (125.77MHz, $CDCl_3$): *δ*C 167.0 and 165.2 (2C=O), 147.9, 147.1, 146.0, 134.8, 130.8, 130.6, 129.9, 128.9, 128.7, 128.2, 127.5, 127.0 and 126.5 (aromatic carbons), 62.5 and 61.4 (2OCH2), 14.1 and 13.5 (2CH3).

Anal. (Calcd. for $C_{21}H_{19}NO_4$: 349.39): C 72.19, H 5.48, N 4.01%. Found: C 70.40, H 5.52, N 3.68%.

MS (Exact mass for $C_{21}H_{19}NO_4$: 349.13): Found $(M+H)^+$ 350.1387.

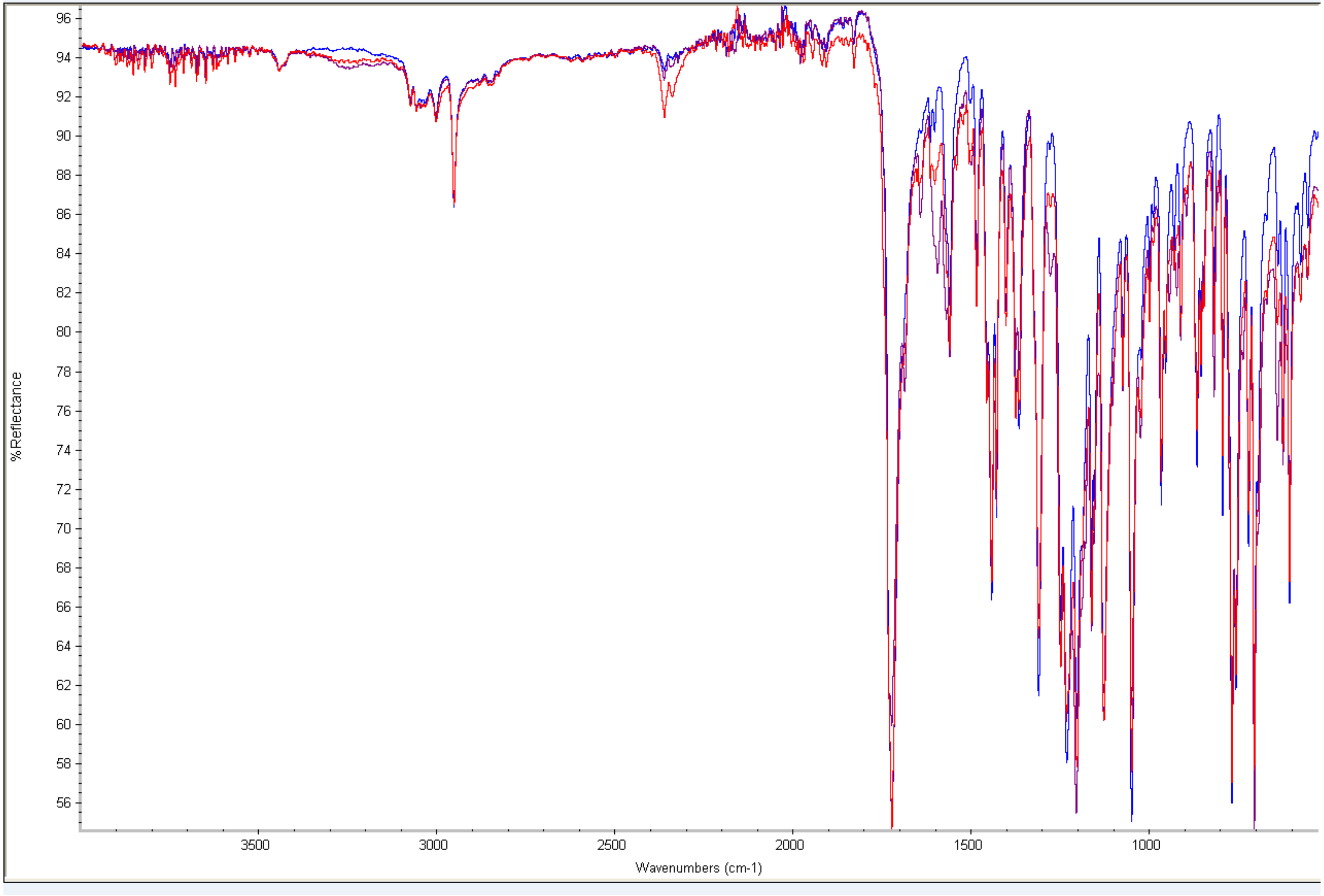

Figure S13: IR spectrum of compound 3g

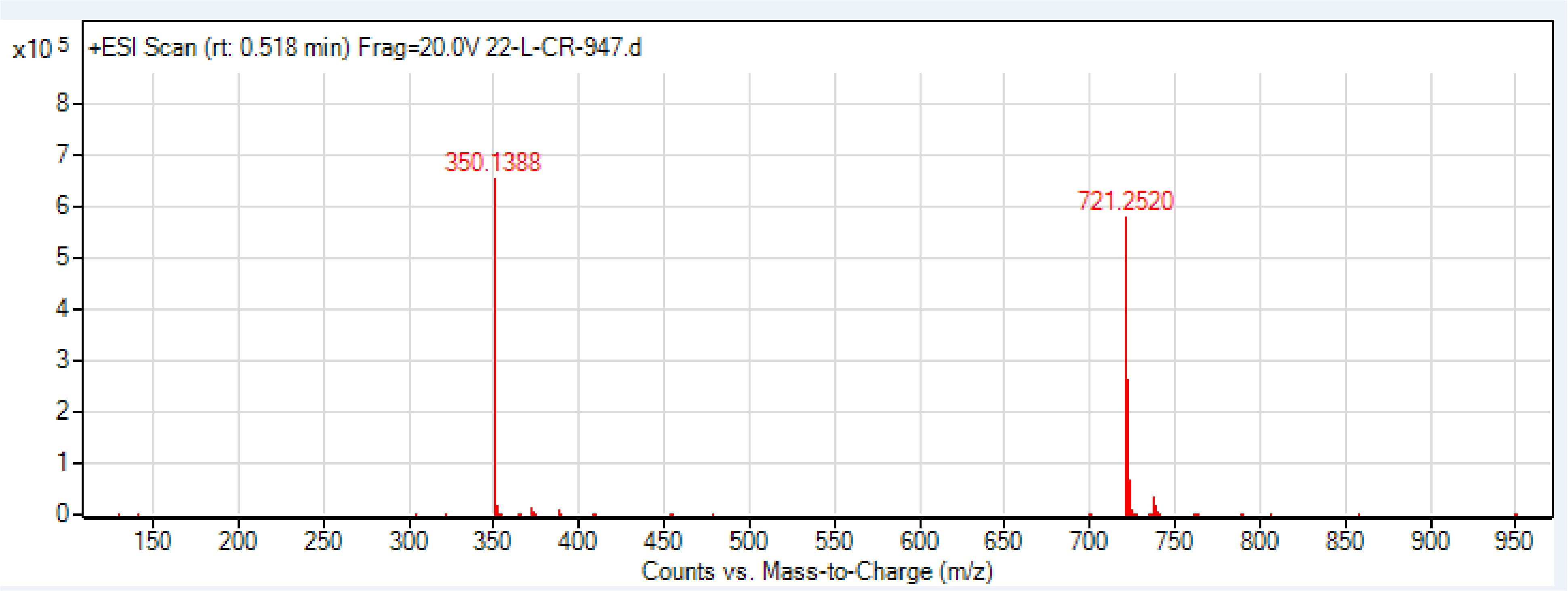

Figure S14: Mass spectrometry for compound 3g

Di-tert-butyl 6-chloro-4-phenylquinoline-2,3-dicarboxylate (3o)

^{1}H NMR (500.13, $CDCl_3$) : *δ*H 8.23 (d, 3 *J*HH = 8.5Hz, 1H, CH), 7.76 (ddd, 3 *J*HH = 8.5Hz,3 *J*HH = 7.8Hz,4 *J*HH = 1.4Hz, 1H, CH), 7.59 (d, 3 *J*HH = 7.8Hz, 1H, CH), 7.53 (t, 3 *J*HH = 7.8Hz, 1H, CH), 7.46 (t, 3 *J*HH = 6.5Hz, 1H, CH), 7.45 (d, 3 *J*HH = 2.2Hz, 2H, 2CH), 7.33 (dd, 3 *J*HH = 6.5Hz,3 *J*HH = 2.2Hz, 2H, 2CH), 4.50 (q, 3 *J*HH =7.1Hz, 2H, OCH2), 4.06 (q, 3 *J*HH = 7.1Hz, 2H, OCH2), 1.43 (t, 3 *J*HH = 7.1Hz, 3H, CH3), 0.96 (t, 3 *J*HH = 7.1Hz, 3H, CH3).
